# Soft Computing for Analysis of Biomedical Data

**DOI:** 10.1155/2018/3902484

**Published:** 2018-11-15

**Authors:** Federico Divina, Miguel García-Torres, Ting Hu, Christian E. Schaerer

**Affiliations:** ^1^Universidad Pablo de Olavide, Seville, Spain; ^2^Memorial University of Newfoundland, St. John's, Canada; ^3^National University of Asuncion, San Lorenzo, Paraguay

Soft computing (SC) techniques can be used to tackle problems characterized by imprecision, uncertainty, and partial truth to achieve tractability and robustness at a low computational cost.

These features represent the main differences between SC and hard computing techniques and provide SC strategies with the ability to deal with ambiguous situations like imprecision and uncertainty. For this reason, SC techniques can obtain approximate solutions to problems which have no known methods to compute an exact solution. The main SC paradigms include fuzzy systems, evolutionary computation, artificial neural computing, metaheuristics, and swarm intelligence.

Those features render SC particularly suitable for analyzing medical data, which is typically characterized by imprecision and the presence of noise. Moreover, SC techniques allow easily integrating human knowledge, which can help achieve better solutions. Biomedical data may be of different nature: texts, images, signals, and so forth, which typically contain a high presence of noise.

The overall aim of this special issue was to compile the latest research and development, up-to-date issues, and challenges in the field of SC and its applications to biomedical data.

Seventeen articles were submitted to this special issue, and finally, six original research articles were accepted and are contained in this special issue.

In the article from Y. X. Meng et al., the authors used functional gene enrichment analysis to identify genetic markers and pathways that are associated with neonatal sepsis. A case-control population based dataset was collected for the purpose of the study, and subsequent statistical tests and coexpression network analysis were employed. A set of 7 key signaling pathways and 7 hub genes were identified with high potential associated with the disease risk.

Biomedical text mining was the subject of the article from A. Onan. In particular, the author proposed an efficient multiple classifier approach to text categorization based on swarm-optimized latent Dirichlet allocation and diversity-based ensemble pruning. The proposed technique was applied to five biomedical text benchmarks. Results showed that the proposed technique outperformed other state-of-the-art classification algorithms, as well as various ensemble learning and ensemble pruning methods.

In article from Y. Li et al., different machine learning techniques were applied to the classification of diabetes follow-up data. In particular, after having applied feature selection and imbalanced processing techniques, the authors applied Support Vector Machine, Decision Trees, Adaboost, and Bagging to the resulting data. Results showed that Adaboost was the most successful technique for classifying this kind of data. Following these results, an analysis of the most relevant features was also conducted.

In their work, B. Yang and coworkers address the problem of studying the neural activity under cognitive reappraisal on simultaneous EEG (electroencephalography)-fMRI (functional magnetic resonance imaging) data. For such a purpose, the authors propose an effective fusion framework that uses a Kernel-based Canonical Correlation Analysis (KCCA). Results show that the proposed EEG-fMRI fusion approach provides an effective way to study the neural activities of cognitive reappraisal with high spatiotemporal resolution.

A novel tool for the optimization of the structure of gene networks is proposed in the article from F. Gómez-Vela et al. In particular, the tool is called GeSOp, and it represents a new computational method for optimizing the structure of gene networks. Such a method performs a pruning of irrelevant information in the input network to facilitate the interpretation of the biological knowledge that comprises the network. To do this, GeSOp relies on a greedy heuristic algorithm that selects only the most relevant relationships and helps to identify the Hubs in the network. The performance of the method was tested in different data sets with satisfactory results in all cases.

Finally, D. Liu et al. propose a cosine similarity measure between hybrid intuitionistic fuzzy sets. In order to study this measure, the authors apply it to medical diagnosis and discuss its relevant properties. Then, based on the proposal, the authors present a decision method for medical diagnosis so that a patient can be diagnosed with the disease according to the values of the cosine similarity measure. This measure is compared with other existing similarity measures. Results show the feasibility and effectiveness of the Cosine similarity measure.

We can conclude that this special issue presents different works using different machine learning techniques applied to biomedical data. As a consequence, this issue can prove to be a valuable tool to gain insights on the state of the art of such a field.

